# UAMC-3203 inhibits ferroptosis and promotes functional recovery in rats with spinal cord injury

**DOI:** 10.1038/s41598-024-70926-1

**Published:** 2024-08-30

**Authors:** Shunli Kan, Sa Feng, Xinyan Zhao, Ziyu Chen, Mengmeng Zhou, Linyan Liu, Haoqiang Zhu, Yuelin Cheng, Xuanhao Fu, Wei Hu, Rusen Zhu

**Affiliations:** 1 Department of Spine Surgery, Tianjin Union Medical Center, Tianjin Medical University, Tianjin, China; 2 Tianjin Institute of Spinal Surgery, Tianjin, China

**Keywords:** Spinal cord injury, UAMC-3203, Ferroptosis, Neuroinflammation, Neuroprotection, Biochemistry, Neuroscience, Physiology

## Abstract

Spinal cord injury (SCI) results in irreversible neurological impairment. After SCI, Ferritinophagy-induced free iron released from ferritin can lead to extensive lipid peroxidation and aggravate neurological damage. NRF2/HO-1 pathway is to endow cells with a protective effect against oxidative stress, and it plays an important role in the transcriptional activation of a series of antioxidant and detoxification genes. UAMC-3203 is a ferrostatin-1(Fer-1) analogue with better solubility and stability, which can more effectively inhibit ferroptosis after SCI. A rat SCI model was constructed, and the recovery of motor function was observed after treatment with UAMC-3203. ELISA was employed to assess the impact of UAMC-3203 on inflammation-related factors, while immunofluorescence was utilized to investigate the influence of UAMC-3203 on neuronal count as well as the activation of astrocytes and microglia/macrophages. Malondialdehyde (MDA) were detected to reflect the level of oxidation products. Western blot analysis was used to measure the level of ferroptosis markers and the expression of NRF2/HO-1. Our findings demonstrate that UAMC-3203 inhibits the production of reactive oxygen species (ROS) and lipid peroxides, preventing ferroptosis and reducing neuronal degeneration. Additionally, UAMC-3203 suppresses astrocyte proliferation and microglia/macrophage activation, as well as the release of ferroptosis-related inflammatory factors. These combined effects contribute to the preservation of spinal cord tissue and the facilitation of motor function recovery. UAMC-3203 maybe inhibit ferroptosis after SCI to promote functional recovery.

## Introduction

 Spinal cord injury (SCI) is one of the most serious traumatic diseases. It has the characteristics of high morbidity, high disability rate and lots of complications. It often leads to reduced or even loss of sensory and motor functions below the injured segment. SCI can cause serious damage to the nervous system, and its repair has been a major problem, which confuses the international medical community^[1]^. In recent years, with the development of social economy and transportation, the incidence of spinal cord injury has shown an obvious upward trend. In the past 30 years, its global prevalence rate has increased from 236 to 1298 cases per million population. The global incidence rate of SCI is estimated to be between 250,000 and 500,000 per year^[2]^.

Ferritinophagy leads to an increase in iron concentration at the injury site, and the free iron released from ferritin leads to extensive lipid peroxidation by inhibiting the cystine/glutamate antiporter (system xc-). The transporter is an antiporter protein, consisting of a catalytic subunit (SLC7A11) and a chaperone subunit (SLC3A2), which can transport glutamate out of the cell and cystine into the cell^[3]^. Cystine transferred into cells is the raw material for glutathione synthesis (GSH). GSH is also a cofactor for the activation of glutathione peroxidase (GPX). GPX is an important enzyme that maintains the redox balance in cells^[4]^. When the activation of GPX is inhibited, the antioxidant capacity of cells is reduced, and the lipid reactive oxygen species in cells are increased, eventually inducing ferroptosis^[5]^. There are eight types of GPX proteins found in mammals, but only GPX4 exhibits the ability to combat lipid peroxidation. GPX4 is an enzyme that contains selenocysteine and relies on glutathione for its function. Through the use of GSH, it can facilitate the conversion of specific lipid hydroperoxides into lipid alcohols. Selenocysteine serves as the active site within GPX4, transitioning between its reduced state (Se-H) and oxidized state (Se-OH) to carry out its biological role. This process can be broken down into three main steps: initially, the reduced GPX4 (GPX4-Se-H) is oxidized by lipid peroxides to form the oxidized GPX4 (GPX4-Se-OH), which in turn reduces the peroxides into alcohols; next, GPX4-Se-OH is reduced by the reducing agent GSH to produce a selenium-glutathione adduct (GPX4-Se-SG); and finally, GPX4-Se-SG reacts with GSH once more, converting back to GPX4-Se-H and generating GSSH^[6]^.

Nuclear factor erythroid2-related factor 2 (Nrf2) is a transcription factor encoded by nuclear factor, erythroid 2-like 2 (NFE2L2) gene and a key factor to maintain and regulate the protein homeostasis system, which regulates the expression of more than 1000 genes through complex signal cascade mode^[7]^. The final transcripts are divided into different groups with different functions, including antioxidant defense, detoxification, inflammatory response, autophagic degradation and metabolism, especially Nrf2 plays an important role in preventing lipid peroxidation and ferroptosis. Heme oxygenase 1 (HO-1) mainly catalyzes the decomposition of heme into ferrous iron, carbon monoxide and biliverdin, and is an important antioxidant enzyme in cells^[8]^.

Ferrostatin-1 is the first generation ferroptosis inhibitor, which can significantly inhibit ferroptosis in vitro. However, due to the instability of plasma and metabolism, its efficacy in vivo is weak^[9]^. Ferroptosis inhibitor UAMC-3203 is a Fer-1 analogue with better solubility and stability^[10]^, which can more effectively inhibit ferroptosis and improve post-resuscitation myocardial dysfunction in a rat model of cardiac arrest^[11]^. To date, its underlying mechanisms of action in SCI remain unclear.

In this study, we demonstrated that the compound UAMC-3203 effectively reduces lipid peroxidation levels and inhibits ferroptosis. It also suppresses astrocyte proliferation and microglia/macrophage activation. Additionally, it inhibits the activation of inflammatory factors associated with these processes, leading to a reduction in spinal cord tissue damage.

## Materials and methods

### Animals and treatments

Female Wistar rats aged 8–10 weeks and weighing 230–240 g were purchased from Beijing Huafukang Biotechnology Co (license no: SCXK (Jing) 2019-008). Prior to experimental treatments, all rats were acclimated for 7 days with free access to food and drink in a controlled environment, which humidity and temperature were controlled in a 12-h diurnal cycle^[12]^. All the experimental procedures were followed international guidelines for the care and use of laboratory animals and approved by the Experimental Animal Ethics Committee of Tianjin People’s Hospital(2023-B09) to minimize the experiment-induced pain, distress, and suffering in rats. All experiments were reported following the ARRIVE guidelines. After the rats were treated with 2–3% isoflurane for 4–5 minutes to induce anesthesia by inhalation, the 10th thoracic vertebral lamina was surgically removed, the corresponding spinal cord was exposed, and fixed under the percussion device. The percussion force was 10 g weight, the percussion height was 25 mm using Stereotaxic Instruments (D01611-002, RWD Life Science Company)^[13]^. After the percussion, local congestion and edema of the injured spinal cord could be seen, the hind limbs of the rats were temporarily spastic and twitched, and after the tail was swinging, both lower limbs were paralyzed, the Basso, Beattie, Bresnahan (BBB) score of the day after injury was 0, indicating that the strike model was successfully established, and then the muscles, fascia, and skin were sutured successively. Rats in the sham group were subjected to laminectomy in the absence of contusion. To prevent infection, each rat was given an intramuscular injection at a dose of 1.6 × 10^5^ U penicillin once daily for 3 days. Manual bladder voiding was performed by applying lower abdominal pressure on the bladder, 2 times daily, until the rats recovered their voluntary urination function^[12]^.

### Drug administration

After surgery, 56 rats were randomly divided into 4 groups. The experiment was divided into sham group, SCI group, SCI + UAMC-3203 group (5 mg/kg), and SCI + UAMC-3203 + Brusatol group (UAMC-3203, 5 mg/kg, Brusatol, 2 mg/kg). UAMC-3203(MedChemExpress, CAS:2271358-65-5), a novel ferroptosis inhibitor, was given intraperitoneal injection 30 min after modeling and then for 7 consecutive days(10; 11). Brusatol (MedChemExpress, CAS:14907-98-3), a NRF2 inhibitor, was given intraperitoneal injection 30 min after modeling and then for 7 consecutive days^[14]^.

A time course for permeability of the injured blood-spinal cord barrier (BSCB) also existed around the lesion site, which started several minutes after SCI, and lasted for up to 28 days after the prime injury. Therefore, we reasonably inferred that intraperitoneal injection of UAMC-3203 could pass the BSCB to a certain extent and achieve therapeutic effects within 7 days after SCI^[15]^.

### Histopathological analysis

Rats were anesthetized and perfused with 0.9% NaCl followed by 4% paraformaldehyde through the heart at week 4 post-Spinal Cord Injury. Approximately 10 mm of spinal cord tissue was dissected from the focal point of the lesion. The samples were then immersed in 4% paraformaldehyde and kept at 4 °C for 24 h. The paraffin sections were incubated in a 60 °C oven for 1.5–2 h, soaked in xylene and alcohol for dewaxing, and rinsed with distilled water. Place the dehydrated paraffin sections in hematoxylin staining solution for 3–5 min and rinse with distilled water. The sections were dehydrated in 85% and 95% graded alcohol for 5 min, and then stained in eosin staining solution for 5 min. The slices were dehydrated and transparent, then air-dried and sealed with neutral gum^[16]^. Luxol Fast Blue(LFB) used LFB kit (G1030,Servicebio, wuhan, China)^[17]^.

### Western blot assay (WB)

Rats were anesthetized with 30 mg/kg pentobarbital sodium (6 mg/mL) three days after spinal cord injury. Tissues located 3 mm from the injury epicenter were collected and lysed in RIPA Lysis Buffer (Beyotime Biotechnology, Cat# P0013B) with added protease inhibitor (Roche, Basel, Switzerland, Cat# 04693132001). The protein concentration was measured by using the BCA assay (P0010,Beyotime, shanghai, China). Target proteins were separated by 12% SDS-PAGE and then transferred to a PVDF membrane. After blocking with 5% skim milk, the membranes were incubated with different primary antibodies at 4 °C overnight. Subsequently, the membranes were washed 3 times with Tris-buffered saline Tween and then incubated with secondary antibodies for 1 h at room temperature. Protein bands were visualized by using ECL reagents (Thermo Scientific, #35050, MA, USA) and exposed by using the ChemiDoc XRS System (BioRad, USA). Quantitative analysis was performed with Image J software^[18]^.

The antibodies were listed as follows: Rabbit Anti-GPX4 antibody (1:2000;Affinity, DF6701,China), Rabbit Anti-ACSL4 (1:10000;Abcam, Ab155282, USA), Rabbit Anti-xCT antibody(1:1000;Abcam, Ab175186, USA), Rabbit Anti-NRF2 antibody(1:2000;Affinity, AF0639,China), Rabbit Anti-HO-1 antibody(1:1500; Affinity, AF5393,China), Mouse Anti-GAPDH (1:1000,GOODHERE BIOTECH, AB-P-R001,Chin), Anti-mouse IgG, HRP-linked Antibody, (1:1000,7076,CST, USA), Anti-rabbit IgG, HRP-linked antibody, (1:1000, 7074, CST, Danvers, MA, United States).

### Enzyme-linked immunosorbent assay (ELISA) analysis

Samples were taken at days 7 after injury. The supernatant was collected from T10 spinal cord tissues. Later, samples and enzymes were added, and incubation, mixing, washing, color development and termination were conducted. Tissue cytokine content was determined following the manufacturer’s instructions with Rat ELISA Kits (F2923, F3056, F-3067, F3071, Fankewei, shanghai, China) for IL-1β, TNF-α, IL-4, and IL-10.

### Malondialdehyde (MDA) and glutathione (GSH) measurement

Rats were anesthetized with 30 mg/kg pentobarbital sodium (6 mg/mL) at 3 days post spinal cord injury (SCI). Tissue samples from the SCI epicenter were collected and homogenized using an automatic TissueLyser (48T, Nanjing Jiancheng Bioengineering Institute, Nanjing, China) for 60 s. The concentrations of MDA in spinal cord tissue were determined according to the manufacturer’s instructions using the Lipid Peroxidation MDA Assay Kit (S0131S, Beyotime Biotechnology, Shanghai, China) ^[19]^. Additionally, the expression of GSH in tissues was detected using the GSH kit (S0053, Beyotime Biotechnology, Shanghai, China) following the manufacturer’s protocol^[20]^.

### Immunofluorescence

The rats were anesthetized with pentobarbital sodium, perfused with PBS and 4% paraformaldehyde (PFA) at 4 ℃ through the heart on the 28th days after the injury. In the context of immunofluorescence staining of spinal cord tissue, the segment containing the injury site was extracted and fixed in 4% paraformaldehyde overnight. Subsequently, the spinal cord tissues underwent dehydration in a 30% sucrose solution for 3 days, followed by embedding in OCT and slicing into 10-µm thick sections. These frozen sections were then permeabilized and blocked before being incubated with primary and secondary antibodies^[18]^. Finally, the tissue sections were visualized using an ultra-high resolution laser confocal microscope (ZEISS LSM 900, Germany).

The used primary/second antibodies are listed as follows: rabbit anti-NeuN (1:500; Abcam, ab177487, USA), rabbit anti- Myelin Basic Protein (1:50, CST, 78896), rabbit anti-GFAP (1:200; Cell Signaling Technology, 3670, USA), rabbit anti- Iba1 (1:200, Proteintech Group, Inc, 10904-1-AP), goat anti-rabbit IgG (1:100, Boster Bio, BA1105, CHAINA).

### Reactive oxygen species (ROS)

ROS levels were measured by the ROS probe dihydroethidium (DHE) fluorescence staining method at day 1 after spinal cord injury. About 10 μm of spinal cord frozen section were washed with PBS at 37 °C for half an hour and replaced with prepared 10 µM DHE (Gene Copoeia) containing PBS, avoided light, at 37 °C for 40 min^[21]^. We observed and photographed under a fluorescence microscope (Zeiss, Heidenheim, Germany, 518 nm).

### Basso, Beattie, Bresnahan (BBB) score

BBB score was utilized to evaluate lower limb motor function in rats following spinal cord injury surgery. The rats were placed in an open field and observed by three independent examiners who were blinded to the groups. The assessments were conducted on days 0, 1, 3, 7, 14, 21, and 28 post-SCI surgery.

### Incline plate test

Nerve function recovery in rats was evaluated through an incline plate test at various time points post-SCI surgery, including days 0, 1, 3, 7, 14, 21, and 28. The experimental procedure involved placing the animals on an inclined plate and recording the maximum angle value achieved within 5 s post-SCI by adjusting the incline. The maximum angle that a normal rat can stay on the inclined plate is 43°. Assessment was carried out by three independent examiners who were blinded to the group information.

### Behavioral evaluation

Gait dynamics were assessed using Catwalk-assisted gait analysis (Noldus Information Technology B.V, Netherlands) on the 28th day post spinal cord injury. The CatWalk system was utilized to test rats in each group, with gait parameters automatically calculated by CatWalk XT 10.6 software. Before formal measurement, the test animals were trained at least five times to obtain reliable results. The performance of each rat moving in one direction without stopping or turning was recorded and repeated three times. Because the recovery of the hind limbs of some rats in the SCI group was insufficient to support the body during walking, dragging occurred after the soles of the feet touched the ground. The study focused on evaluating the impact of specific gait parameters, such as max Contact Area, regularity index, and stands (stop time), on behavioral changes following spinal cord injury. An electrophysiological device (YRKJ-G2008; Zhuhai Yiruikeji Co, Ltd, Guangdong, China) was utilized to assess motor evoked potential (MEP) in rats at the four-week mark post spinal cord injury (SCI) in order to assess the restoration of nerve conduction function^[18]^.

### Statistical analysis

All data were presented as mean ± standard deviation (SD). One-way analysis of variance (ANOVA) was performed to compare multiple groups, which were followed by Tukey multiple comparison post hoc test. Unpaired Student’s t-test was used to compare two groups using SPSS Statistics 25.0 (SPSS Inc., Chicago, IL, USA). Statistical significance was defined as: * means *p* < 0.05, ** means *p* < 0.01, and *** means *p* < 0.001.

## Results

### UAMC-3203 promotes the recovery of motor function after SCI in rats

**Fig. 1:**
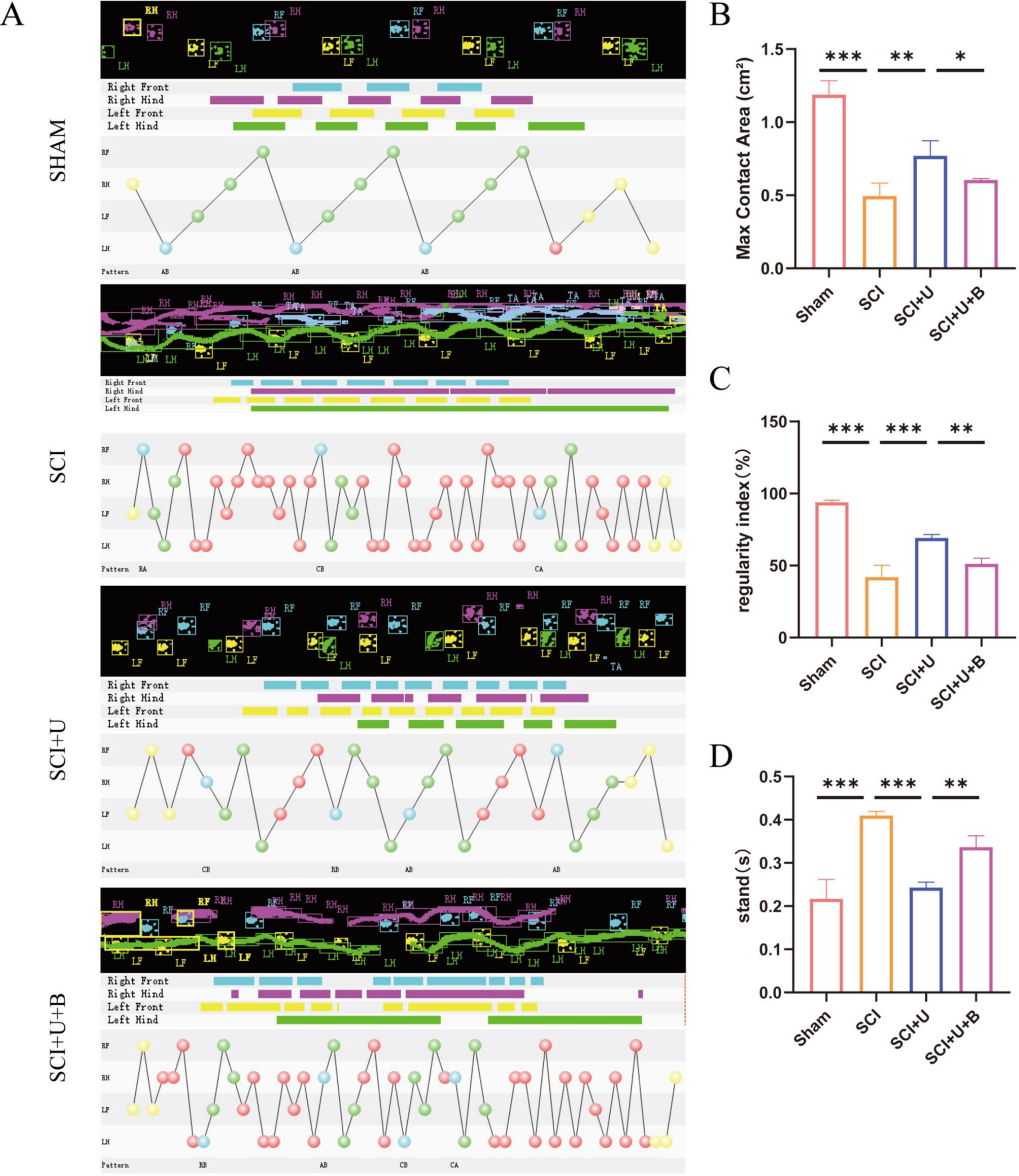
UAMC-3203 promotes the recovery of the hind limbs in spinal cord injured rats. **(A)** Representative paw step images and limbs’ supporting timing view of CatWalk gait analysis. **(B-D)** Quantitative analysis of catwalk at day 28 post-injury, including max contact area, regularity index, and stands (stop time) (*n* = 3). Data were presented as mean ± standard deviation. Results were analyzed by One-way ANOVA. * means *p* < 0.05, ** means *p* < 0.01, and *** means *p* < 0.001. SCI: spinal cord injury; SCI + U: spinal cord injury + UAMC-3203; SCI + U + B: spinal cord injury + UAMC-3203 + Brusatol.

**Fig. 2:**
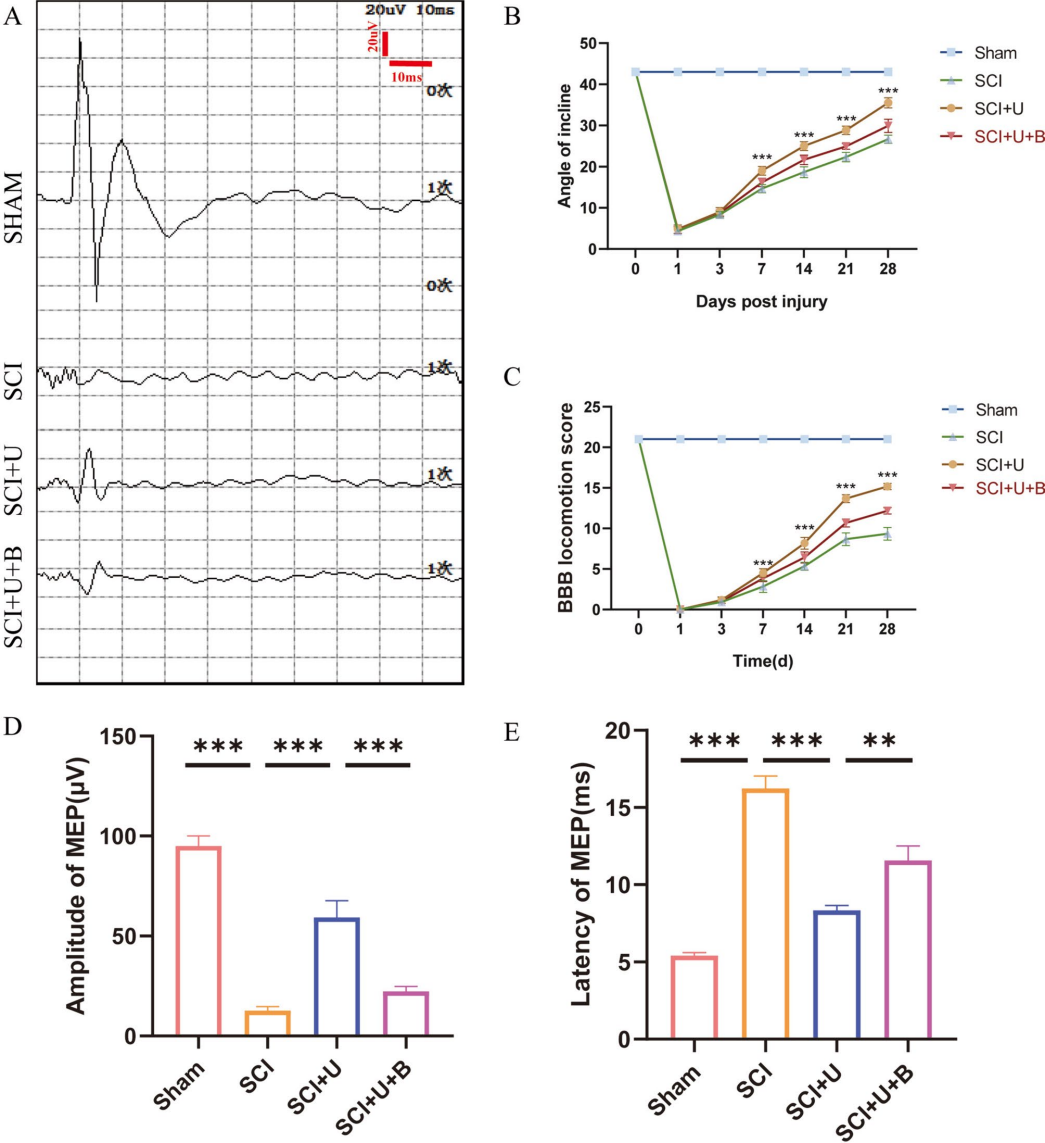
UAMC-3203 promotes the recovery of motor function after SCI in rats. **(A)** Motor evoked potential (MEP) was performed as an electrophysiological assessment in sham group, SCI group, SCI + U group and SCI + U + B group at day 28 post-injury. **(B)** Use inclined plate test to evaluate the recovery of motor function in sham group, SCI group, SCI + U group and SCI + U + B group, respectively, in 1, 3, 7, 14, 21, 28 days (*n* = 12). **(C)** Use BBB score to evaluate the recovery of motor function in sham group, SCI group, SCI + U group and SCI + U + B group, respectively, in 1, 3, 7, 14, 21, 28 days (*n* = 12). **(D-E)** Quantitative analysis of MEP at day 28 post-injury, including Amplitude and Latency (*n* = 3). Data were presented as mean ± standard deviation. Results were analyzed by One-way ANOVA. * means *p* < 0.05, ** means *p* < 0.01, and *** means *p* < 0.001. SCI: spinal cord injury; SCI + U: spinal cord injury + UAMC-3203; SCI + U + B: spinal cord injury + UAMC-3203 + Brusatol.

To assess the motor function of the hind limbs in spinal cord injured rats, Catwalk footprint analysis was applied. The results indicated that the SCI + U group showed better gait coordination 28 days after surgery than the SCI group, especially in the max contact area, regularity index, and stands (stop time) (Fig. 1A and D). To assess the improvement in nerve conduction, electrophysiological assay was performed on rats. The MEP results showed that the MEP amplitude increased and the MEP Latency decreased in the SCI + U group compared to the SCI group (Fig. 2A, D and E). In order to study the effect of UAMC-3203 on the functional recovery of rats with SCI, BBB scores and inclined plate test were used to evaluate the motor function of rats on the 1st, 3rd, 7th, 14th, 21st and 28th days after surgery. Rats in the sham group exhibited a BBB motor score of 21 at each postoperative time point (1, 3,7, 14, and 28 days), with no evidence of hindlimb motor dysfunction. Conversely, rats in SCI, SCI + U and SCI + U + B groups exhibited severe motor dysfunction at 1 d post-operation, as evidenced by a score close to 0. It continued to rise during the experimental period but remained significantly lower in the SCI group and SCI + U than in the sham group at all time points. Rats in the SCI + U group exhibited significantly higher BBB scores at 7,14, 21, and 28 d after SCI, relative to their SCI counterparts. The BBB score of the SCI + U + B group was significantly lower than that of the SCI + U group at 14, 21, and 28 days after spinal cord injury (Fig. 2C). The inclined plate test also confirmed that the motor score of the SCI + U group was significantly improved compared with the SCI (Fig. 2B). In short, the above experiments show that after SCI, UAMC-3203 treatment can promote the recovery of motor function after SCI in rats.

### UAMC-3203 upregulates NRF2 and HO-1 proteins and inhibits the production of ROS after SCI in rats

**Fig. 3:**
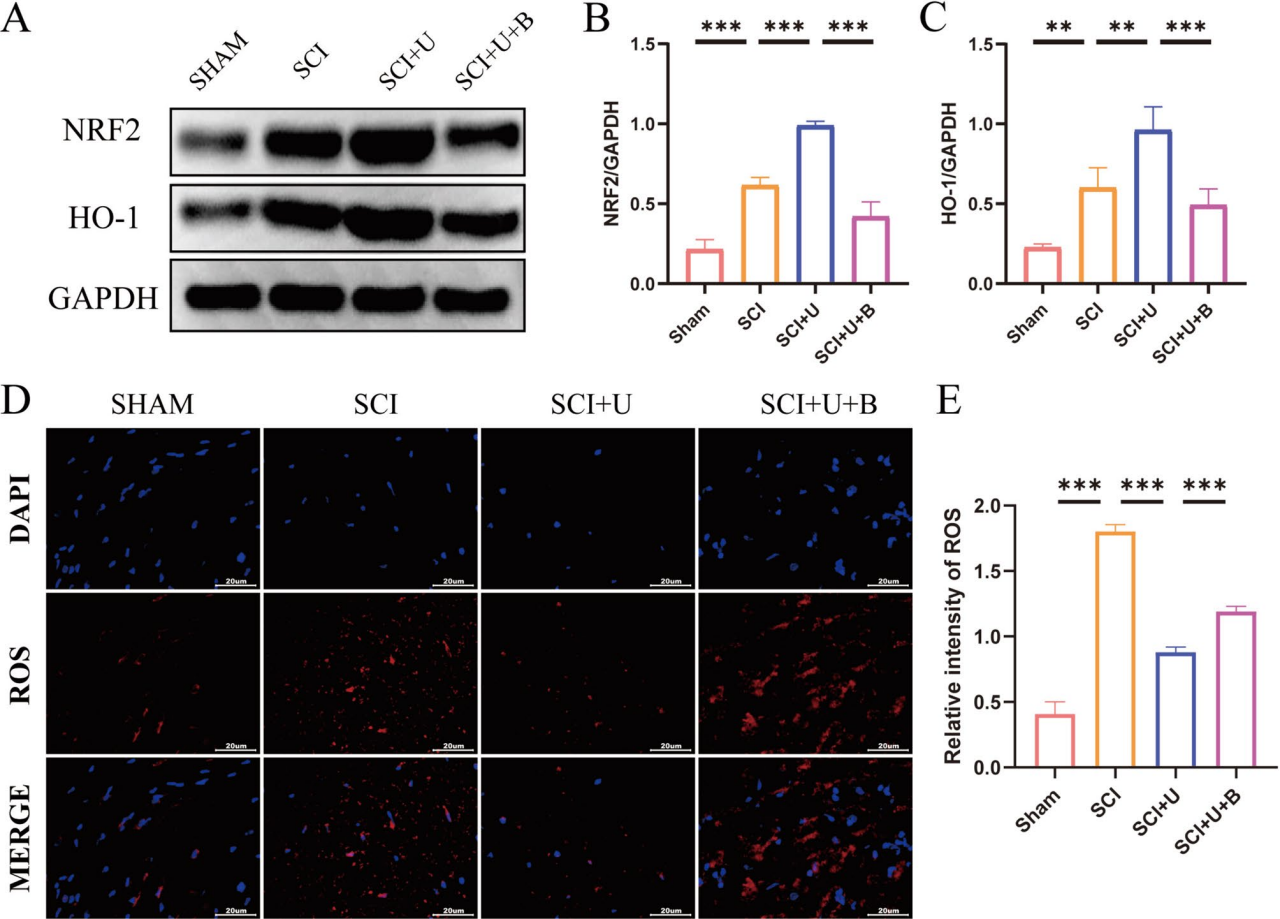
UAMC-3203 upregulates NRF2 and HO-1 proteins and inhibits the production of ROS (reactive oxygen species) after SCI in rats. (A) The expressions of NRF2 and HO-1 were detected by Western blot assays on day 3 after SCI. (B-C) Quantitative analysis of western blot assays (*n* = 3). (D-E) DHE (dihydroethidium) staining of spinal cord tissue on day 1 after SCI and fluorescence quantitative analysis (*n* = 3). Scale bar: 20 μm. Data were presented as mean ± standard deviation. Results were analyzed by One-way ANOVA. * means *p* < 0.05, ** means *p* < 0.01, and *** means *p* < 0.001. SCI: spinal cord injury; SCI + U: spinal cord injury + UAMC-3203; SCI + U + B: spinal cord injury + UAMC-3203 + Brusatol.

Hemorrhage of the spinal cord leads to increased iron concentration at the injury site, then iron-mediated Fenton reaction generate more ROS ^[5]^. The NRF2 signaling pathway is a direct downstream pathway of ROS, which regulates the transcription of ARE-dependent genes to balance oxidative mediators and maintain cell redox homeostasis ^[7]^. The WB results demonstrated that SCI led to an increase in the expression of NRF2 and HO-1 proteins compared to the SHAM group. Furthermore, UAMC-3203 treatment further enhanced the levels of NRF2 and HO-1 proteins when compared to the SCI group. Notably, Brusatol, a NRF2 inhibitor, reversed the elevated expression of NRF2/HO-1 protein induced by UAMC-3203 (Fig. 3A and C). DHE staining revealed a significant increase in ROS levels in the SCI group compared to the SHAM group. However, the SCI + U group effectively reduced the expression of ROS compared to the SCI group. Interestingly, the addition of Brusatol reversed the ROS-reducing effect of UAMC-3203 (Fig. 3D and E). Collectively, these findings suggest that UAMC-3203 upregulates NRF2 and HO-1 proteins and inhibits ROS.

### UAMC-3203 reduces lipid peroxides after SCI

**Fig. 4:**
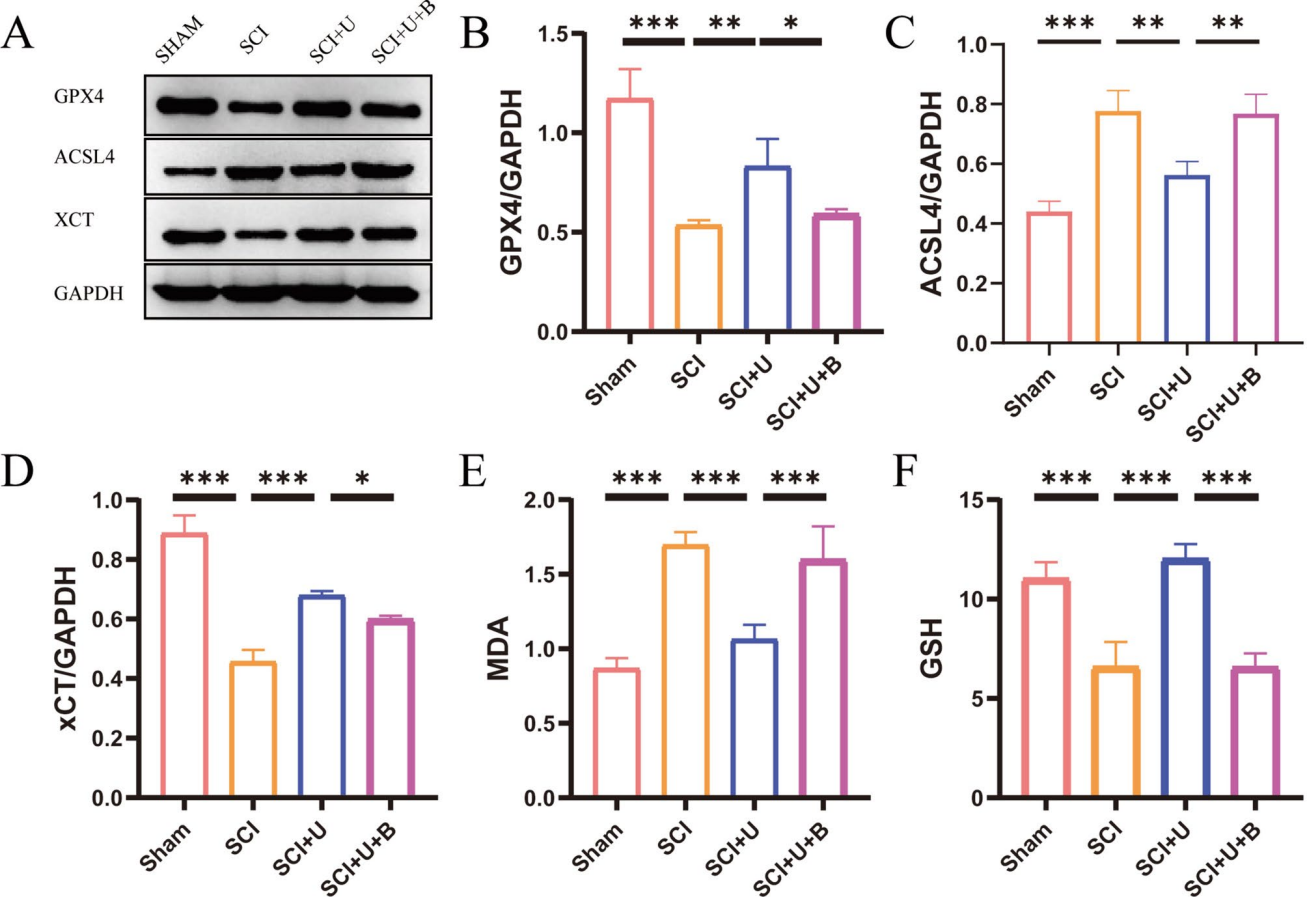
UAMC-3203 can reduce the ferroptosis markers of damaged parts and lipid peroxide levels after SCI. (A) The expressions of GPX4 (glutathione peroxidase 4), ACSL4 (Acyl-CoA synthetase long-chain family member 4), and XCT (glutamate/cystine reverse transporter system) were detected by Western blot assays three days after spinal cord injury. (B-D) Quantitative analysis of western blot assays (*n* = 3). (E-F) The concentration of GSH (glutathione synthesis) and MDA (malondialdehyde) in sham group, SCI group, SCI + U group, and SCI + U + B (*n* = 9) at 3 days post spinal cord injury. Data were presented as mean ± standard deviation. Results were analyzed by One-way ANOVA. * means *p* < 0.05, ** means *p* < 0.01, and *** means *p* < 0.001. SCI: spinal cord injury; SCI + U: spinal cord injury + UAMC-3203; SCI + U + B: spinal cord injury + UAMC-3203 + Brusatol.

Severe free radical reactions originated from fenton reaction was believed to trigger ferroptosis by facilitating the formation of oxidation of membrane phospholipids. Lipid peroxidation seems to be a hallmark of ferroptosis^[22]^. To explore whether ferroptosis-related proteins were changed, WB was performed. GPX4 and xCT showed a noticeable decrease, and ACSL4 increased significantly in the SCI group. Furthermore, the application of UAMC-3203 effectively ameliorated the down-regulation of GPX4 and xCT and the up-regulation of ACSL4 caused by spinal cord injury. SCI + U group expressed more GPX4, xCT and less Acsl4 than SCI + U + B group (Fig. 4A and D). The results of lipid peroxidation analysis revealed an increase in MDA expression and a decrease in GSH expression after SCI. However, UAMC-3203 effectively inhibited the increase in MDA and the decrease in GSH. Furthermore, when Brusatol was added, the expression of MDA increased and the expression of GSH decreased (Fig. 4E and F). In summary, UAMC-3203 can inhibit lipid peroxidation and ferroptosis after SCI, which may be related to UAMC-3203 upregulating NRF2 and HO-1 proteins to reduce ROS production. Brusatol, a NRF2 inhibitor, blocks the positive effects of UAMC-3203 in rats after SCI.

### UAMC-3203 reduces the damage of spinal cord tissue and motor neuron loss after SCI

**Fig. 5:**
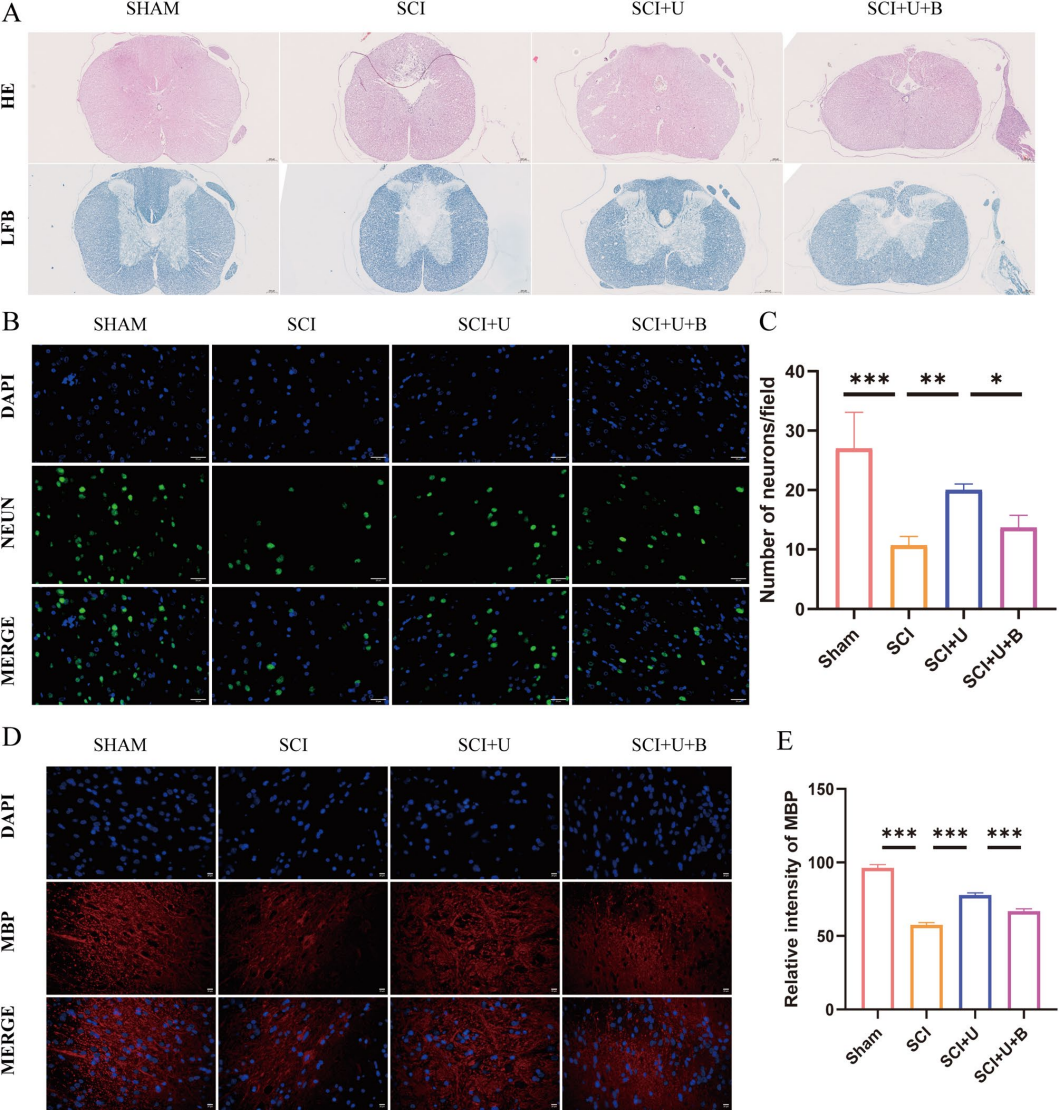
UAMC-3203 reduces the damage of spinal cord tissue and motor neuron loss after SCI. (A) HE-stained and LFB-stained transverse sections of spinal cords from sham group, SCI group, SCI + U group, and SCI + U + B group 28 days after the injury. Scale bar: 200 μm. (B) Use NeuN staining to evaluate the number of neuronal cells in sham group, SCI group, SCI + U group, and SCI + U + B group 28 days after the injury. Scale bar: 20 μm. (C) Quantitative analysis of immunofluorescence assays (*n* = 3). (D) Use MBP (myelin Basic protein) staining to evaluate the undamaged myelin in sham group, SCI group, SCI + U group, and SCI + U + B group 28 days after the injury. Scale bar: 20 μm. (E) Quantitative analysis of immunofluorescence (*n* = 3). Data were presented as mean ± standard deviation. Results were analyzed by One-way ANOVA. * means *p* < 0.05, ** means *p* < 0.01, and *** means *p* < 0.001. SCI: spinal cord injury; SCI + U: spinal cord injury + UAMC-3203; SCI + U + B: spinal cord injury + UAMC-3203 + Brusatol.

Subsequently, we used HE staining and NeuN staining for histological analysis of spinal cord tissue. We also observed myelin preservation, which was another pivotal factor for locomotion recovery after SCI. LFB staining and the fluorescence of myelin Basic protein (MBP) were performed to assess the myelin loss. On the 28th day after SCI, HE staining showed that the injured area of ​​rats in the SCI group was larger than that of the SHAM group, and the tissue structure of the SCI + U group was significantly improved. Data of LFB staining showed that SCI significantly decreased myelin at the injury site (Fig. 5A). NeuN staining showed that the number of neurons around the spinal cord injury area in the SCI group was significantly reduced, and the UAMC-3203 treatment showed more surviving neuronal cells than the SCI group and the SCI + U + B group (Fig. 5B and C). Moreover, the fluorescence intensity of myelin Basic protein (MBP) further confirmed that the undamaged myelin was significantly increased after UAMC-3203 treatment (Fig. 5D and E). These data indicate that UAMC-3203 treatment can reduce the damage of spinal cord tissue, and promote myelin sheath preservation and neuronal cell survival after SCI in rats.

### UAMC-3203 reduces neuroinflammation and activation of astrocytes and microglia/macrophages in rats after SCI

**Fig. 6:**
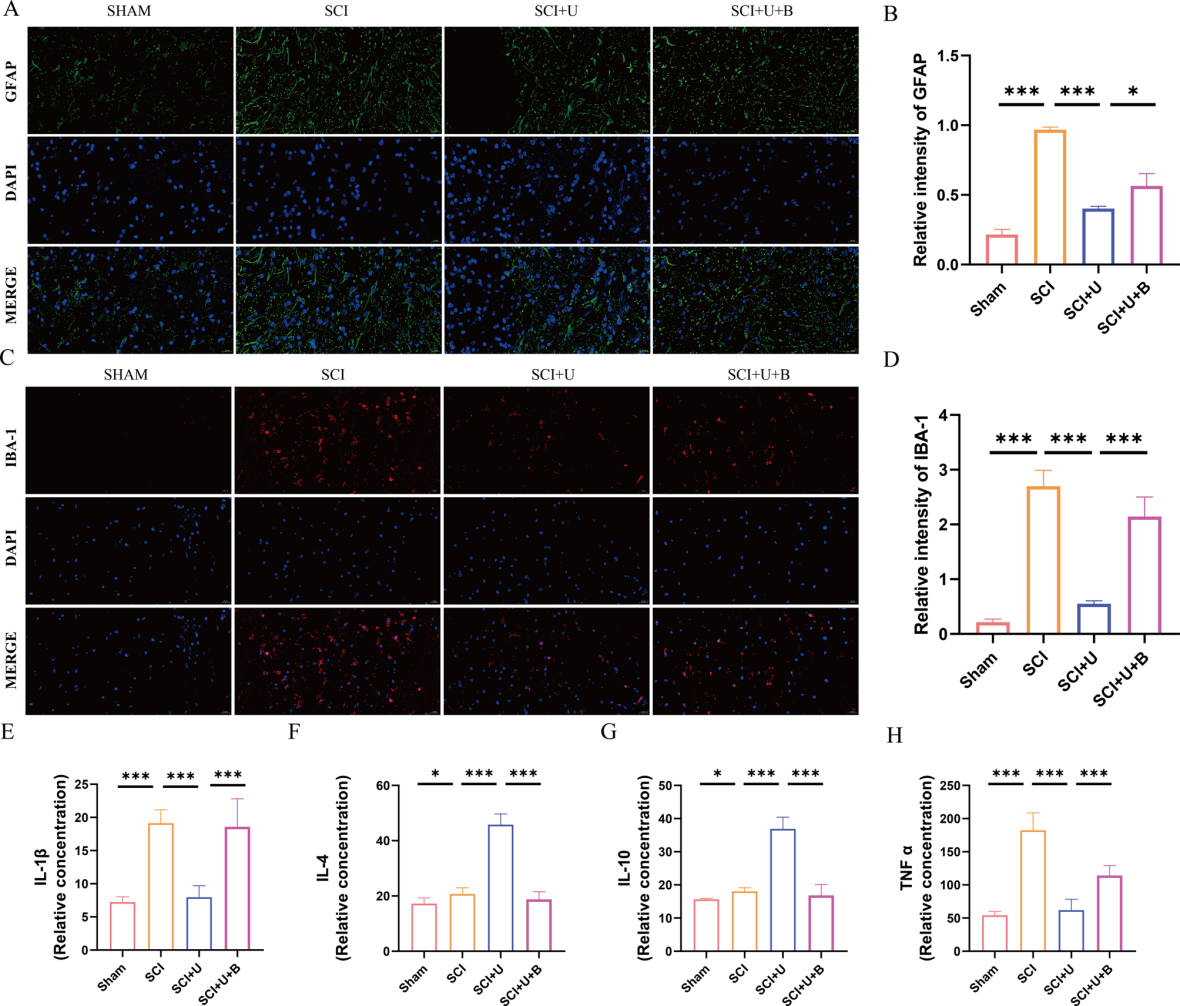
UAMC-3203 reduces neuroinflammation and activation of astrocytes and microglia in rats after SCI. **(A)** Use GFAP staining to evaluate activation of astrocytes in sham group, SCI group, SCI + U group, and SCI + U + B group 28 days after the injury. Scale bar: 20 μm. **(B)** Quantitative analysis of immunofluorescence assays (*n* = 3). **(C)** Use IBA-1 staining to evaluate activation of microglia in sham group, SCI group, SCI + U group, and SCI + U + B group 28 days after the injury. Scale bar: 20 μm. **(D)** Quantitative analysis of immunofluorescence assays (*n* = 3). **(E-H)** The concentration of IL-1β, IL-4, IL-10 and TNFα in sham group, SCI group, SCI + U group, and SCI + U + B (*n* = 9) 7 days after the injury. Data were presented as mean ± standard deviation. Results were analyzed by One-way ANOVA. * means *p* < 0.05, ** means *p* < 0.01, and *** means *p* < 0.001. SCI: spinal cord injury; SCI + U: spinal cord injury + UAMC-3203; SCI + U + B: spinal cord injury + UAMC-3203 + Brusatol.

Studies have shown that ferroptosis is closely related to inflammation, and the process of ferroptosis is often accompanied by inflammation, which is called ferroptosis-related inflammation ^[23]^. GFAP and IBA-1 are critical markers of astrocytes and microglia/macrophages. The immunofluorescence analysis results showed that the fluorescence intensity of GFAP and IBA-1 markedly increased after SCI, and that in SCI + U group was markedly lower compared to the SCI group and SCI + U + B group (Fig. 6A and D). To verify the effect of UAMC-3203 on inflammatory factors, ELISA was performed to detect the releases of IL-1β, TNF-α, IL-4, and IL-10 in spinal cord tissues. Compared with the SCI group, the levels of TNF-α and IL-1β decreased in the SCI + U group, and the anti-inflammatory cytokine, IL-4 and IL-10 increased. However, Brusatol alleviated the effect of UAMC-3203, which inhibited inflammatory factors and increased the effect of anti-inflammatory factors. (Figure 6E and H). In a word, UAMC-3203 reduces neuroinflammation and activation of astrocytes and microglia/macrophages in rats after SCI.

## Discussion

Spinal cord injury (SCI) results in irreversible tissue loss and neurological dysfunction (23; 24). Following SCI, neurons, blood vessels, and glial cells are immediately subjected to mechanical damage. Within hours to days post-SCI, a cascade of secondary injury events unfolds, including increased blood-spinal cord barrier permeability, ion imbalances, edema, glutamate excitotoxicity, lipid peroxidation, and inflammation ^[25]^. This study presents novel findings demonstrating that UAMC-3203 exerts a protective effect in SCI by inhibiting ferroptosis and related inflammatory processes.

Ferrostatin-1 (Fer-1) is a first-generation ferroptosis inhibitor that has demonstrated significant efficacy in vitro ^[9]^. Research has indicated that Fer-1 has the ability to reverse acrylamide-induced damage to dorsal root ganglion neurons by inhibiting ferroptosis ^[26]^. By directly inhibiting lipid peroxidation through the capture of chain-carrying radicals, particularly within the phospholipid bilayer, Fer-1 helps prevent cell death resulting from depletion of GPX4 and GSH. ^[27]^. However, due to the instability of plasma and metabolism, its efficacy in vivo is weak. Iron death inhibitor UAMC-3203 is a Fer-1 analogue with better solubility and stability, which can quickly insert phospholipid bilayer. Rats were intravenously administered 5 mg/kg of UAMC-3203, resulting in plasma concentrations below the detection limit after 6 h, indicating rapid distribution to various tissues^[10]^. Substantial levels of UAMC-3203 were detected in the liver, kidneys, and lungs after 39 h, suggesting improved solubility and stability^[10]^. Toxicity tests revealed no adverse effects in rats following daily injections of UAMC-3203 for 4 weeks ^[10]^. Studies have shown UAMC-3203 improved post-resuscitation myocardial dysfunction through suppressing ferroptosis in a rat model of cardiac arrest by intraperitoneal injection ^[11]^. Catwalk footprint analysis, MEP, BBB scale and inclined plate test were classic methods to evaluate neurological functional recovery in SCI rats^[28]^. In this study, we confirmed for the first time that UAMC-3203 could effectively improve the gait, electrophysiological signal conduction, and motor function induced by SCI. Tissue necrosis and neuron loss induced by SCI were relieved by UAMC-3203.

Ferroptosis, a form of regulated cell death ^[29]^, was initially explored as a potential target for anti-cancer therapy^[26]^. It involves iron-dependent membrane lipid peroxidation, with Acyl-CoA synthetase long-chain family member 4 (ACSL4) playing a key role in this process. Additionally, the glutamate/cystine reverse transporter system Xc– (xCT) and glutathione peroxidase 4 (Gpx4) are crucial components of GSH metabolism. The dehydrogenation of polyunsaturated fatty acids results in the production of phospholipid hydroperoxides, which are then converted into malondialdehyde (MDA), serving as markers for ferroptosis ^[30]^. Ferroptosis can be triggered by a reduction in the function of Gpx4 ^[31]^. When the antioxidant capacity of cells diminishes, reactive oxygen species (ROS) accumulate within the cells, ultimately leading to oxidative cell death. Research has shown that ferroptosis is associated with various conditions such as tumors, ischemia-reperfusion injury, neurodegenerative diseases, and other ailments. Modulation of ferroptosis can either promote or inhibit the progression of these diseases ^[27]^. In our study, we found weaker fluorescence of ROS, downregulated expression of ACSL4 and lower level of MDA in response to UAMC-3203. we found the markedly augmentated GSH and upregulated protein of xCT and Gpx4 in the SCI + U groups.

Numerous studies have demonstrated that the activation of NRF2 can effectively mitigate the damage caused by ferroptosis ^[32]^. NRF2 is widely recognized as the primary regulator of cellular defense mechanisms against injury(33; 34). Its primary role is to confer cells with a protective shield against oxidative stress by orchestrating the transcriptional activation of a range of antioxidant and detoxification genes^[35]^. When cells experience oxidative stress, the interaction between NRF2 and Keap1 in the cytoplasm is disrupted ^[36]^. Subsequently, NRF2 translocates to the nucleus where it engages with the antioxidant response element (ARE), culminating in the transcriptional upregulation of vital cell defense genes such as phase II detoxification enzymes like HO-1 and NQO1, along with direct ROS scavenging proteins like GPX, SOD, and CAT (33; 34). HO-1 primarily facilitates the breakdown of heme into ferrous iron, carbon monoxide, and biliverdin. As a result, HO-1 has the properties of vasodilation, anti-inflammation, anti-apoptosis, antioxidant and cell protection^[8]^.

Neuroinflammation is a significant response of spinal cord injury (SCI) to trauma ^[37]^. Studies have shown that ferroptosis can enhance PTGS2 expression and release, which in turn accelerates the metabolism of arachidonic acid and facilitates the release of inflammatory signaling molecules. The initial cells involved in the inflammatory response include microglia, macrophages, and neutrophils ^[23]^, which can lead to tissue damage by releasing pro-inflammatory mediators such as IL-1β and TNF-α^[25]^. Our study showed that the levels of IL-1β and TNF-α were reduced after UAMC-3203 treatment. In addition, immunofluorescence staining also showed that UAMC-3203 could inhibit the accumulation of GFAP-positive glial scars and IBA-1 microglia/macrophages in spinal cord injury areas.

There are several limitations to our study. This research is mainly focused on the effect of UAMC-3203 in rats without demonstrating it on primary neuronal cells. In addition, the dose of UAMC-3203 used (5 mg /kg) is determined according to literature reports.

## Conclusion

In conclusion, we examined the effects of UAMC-3203 on preventing SCI-induced ferroptosis in a rat SCI model. UAMC-3203 inhibited the production of active ROS, decreased lipid peroxidation levels, inhibited ferroptosis, reduced spinal cord tissue damage and motor neuron loss, mitigated the inflammatory response post-spinal cord injury, and enhanced the restoration of motor function in rats. These findings provide new insights into the neuroprotective effect of UAMC-3203 in SCI, UAMC-3203 makes it a potential therapeutic strategy for clinical improvement of SCI.

## Supplementary Information

Below is the link to the electronic supplementary material.

Supplementary Figures.

## Data Availability

Availability of data and materialsThe data used to support the results of this study can be found in this article.
