# Author Correction: UAMC-3203 inhibits ferroptosis and promotes functional recovery in rats with spinal cord injury

**DOI:** 10.1038/s41598-024-78546-5

**Published:** 2024-11-14

**Authors:** Shunli Kan, Sa Feng, Xinyan Zhao, Ziyu Chen, Mengmeng Zhou, Linyan Liu, Haoqiang Zhu, Yuelin Cheng, Xuanhao Fu, Wei Hu, Rusen Zhu

**Affiliations:** 1 Department of Spine Surgery, Tianjin Union Medical Center, Tianjin Medical University, Tianjin, China; 2 Tianjin Institute of Spinal Surgery, Tianjin, China

Correction to: *Scientific Reports* 10.1038/s41598-024-70926-1, published online 30 August 2024

The original version of this Article, Affiliation 1 was incorrect.

“Department of Spine Surgery, Tianjin Union Medical Center, Tianjin, China.”

now reads

“Department of Spine Surgery, Tianjin Union Medical Center, Tianjin Medical University, Tianjin, China.”

The original Article has been corrected.

