# Supplementary material for: UAMC-3203 inhibits ferroptosis and promotes functional recovery in rats with spinal cord injury
Source: Sci Rep. 2024 Aug 30;14:20180. doi: 10.1038/s41598-024-70926-1 (PMC11364804; doi:10.1038/s41598-024-70926-1)

NRF2

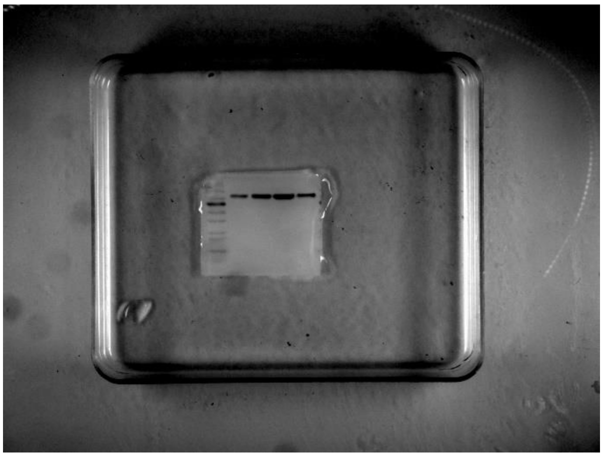

HO-1

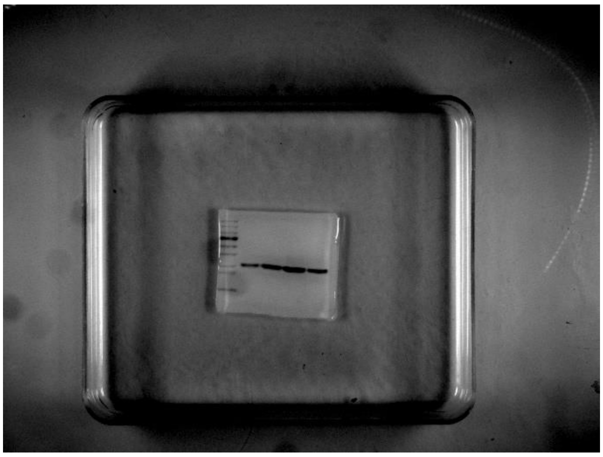

GAPDH

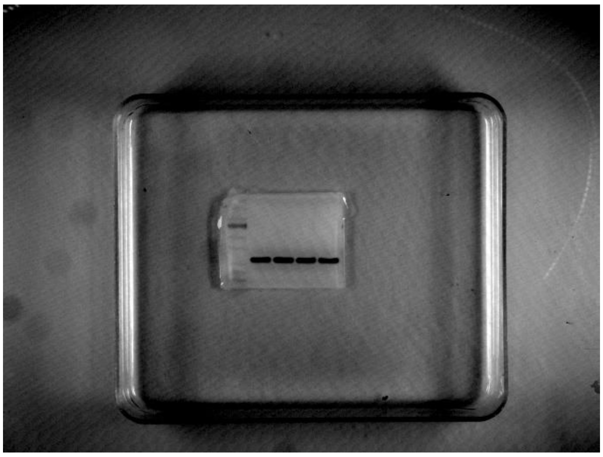

Marker

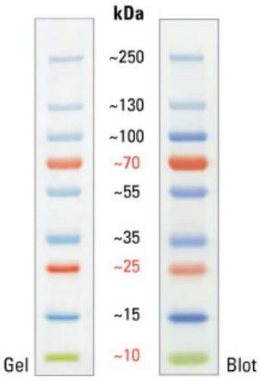

Blue Plus® II Protein Marker

(14-120 kDa)

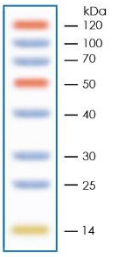

12% Tris-Glycine SDS gel (5 μl/well)

NRF2

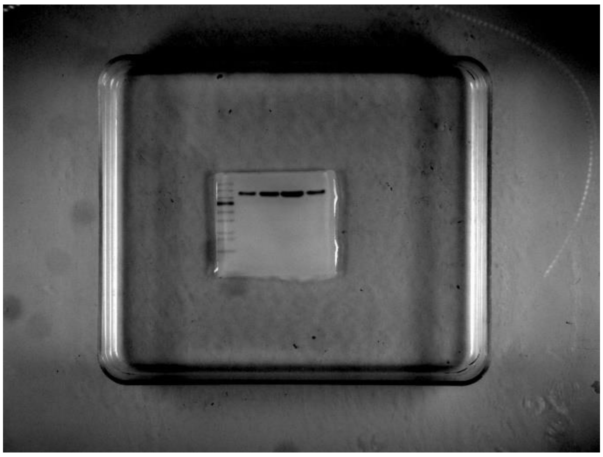

HO-1

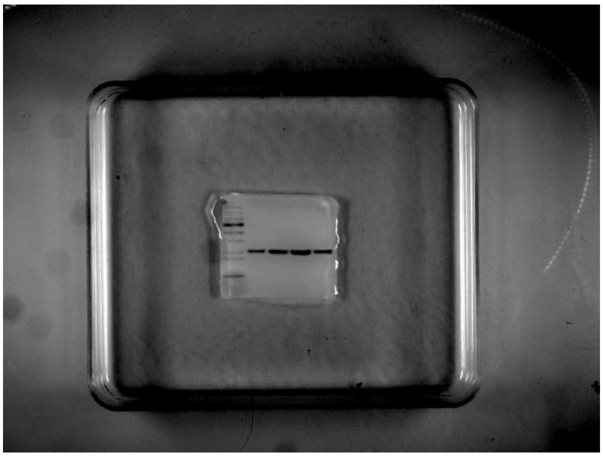

GAPDH

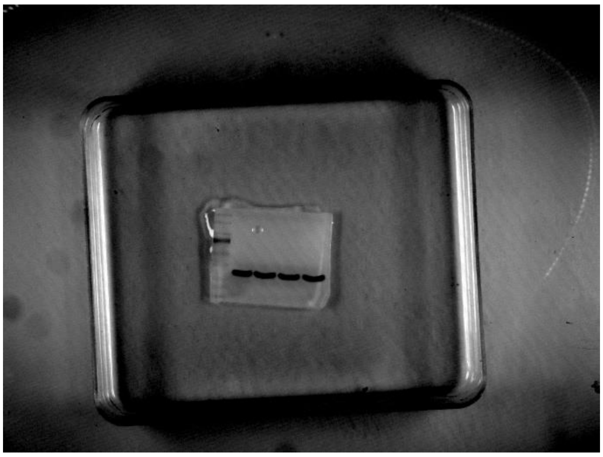

Marker

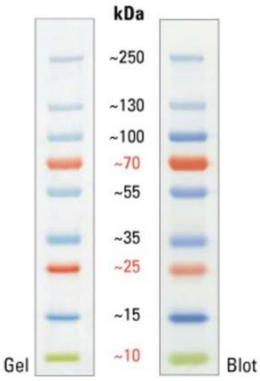

Blue Plus® II Protein Marker

(14-120 kDa)

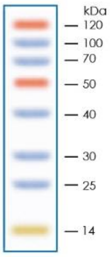

12% Tris-Glycine SDS gel (5 μl/well)

NRF2

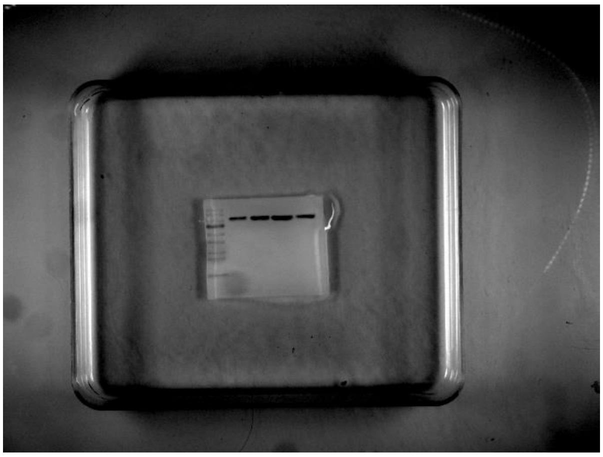

HO-1

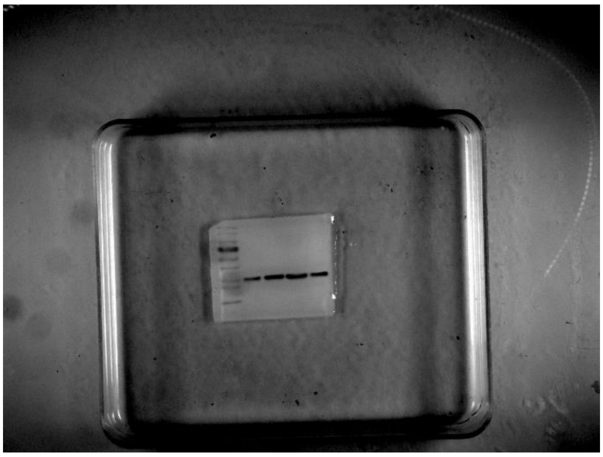

GAPDH

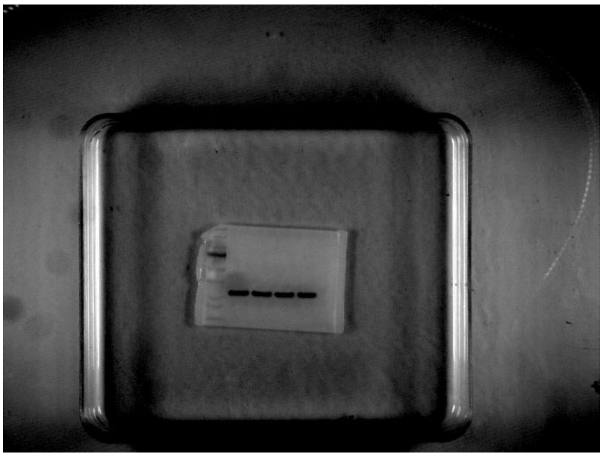

Marker

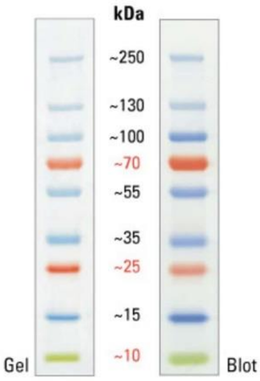

Blue Plus® II Protein Marker
(14-120 kDa)

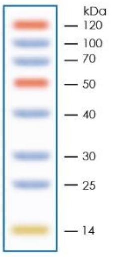

12% Tris-Glycine SDS gel (5 µl/well)

GPX4

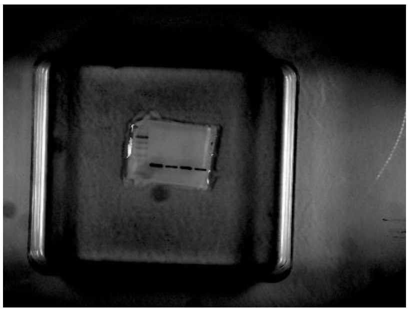

ACSL4

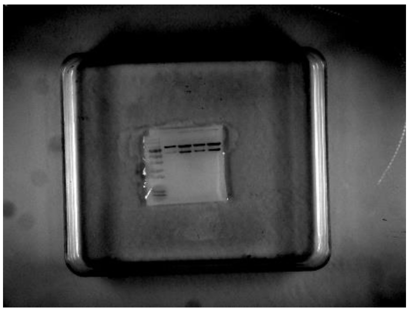

XCT

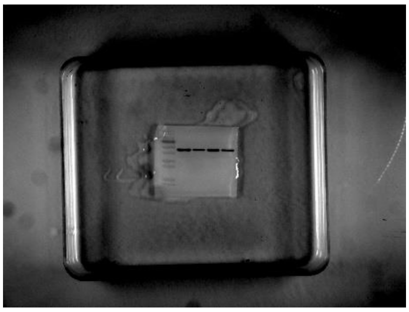

GAPDH

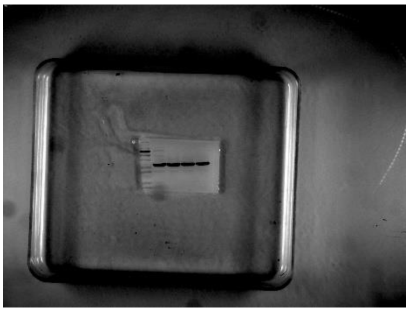

Marker

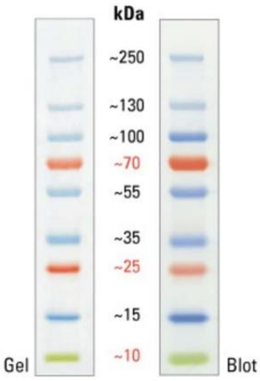

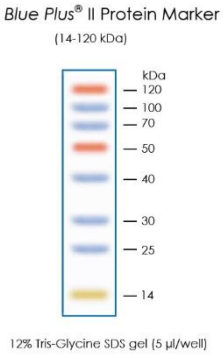

GPX4

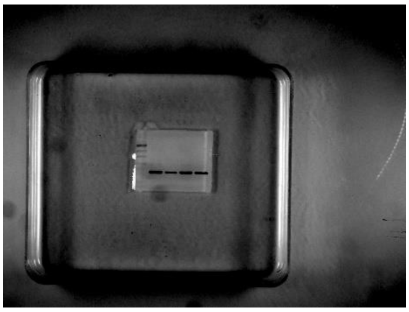

ACSL4

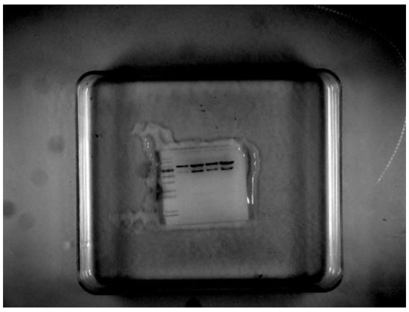

XCT

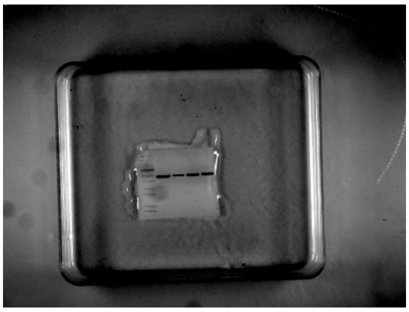

GAPDH

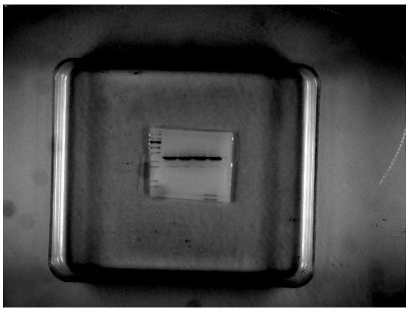

Marker

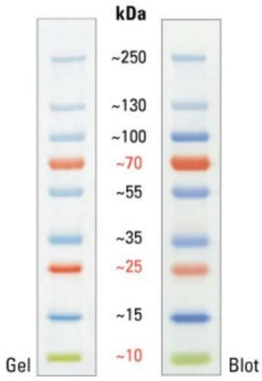

Blue Plus® II Protein Marker
(14-120 kDa)

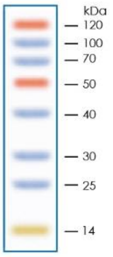

12% Tris-Glycine SDS gel (5 μl/well)

GPX4

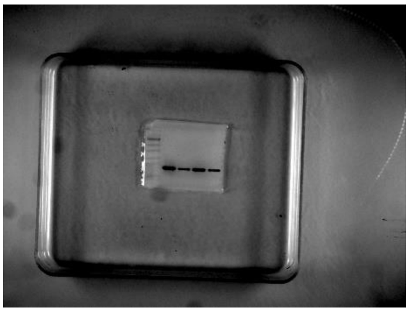

Marker

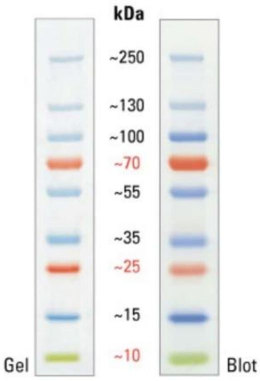

XCT

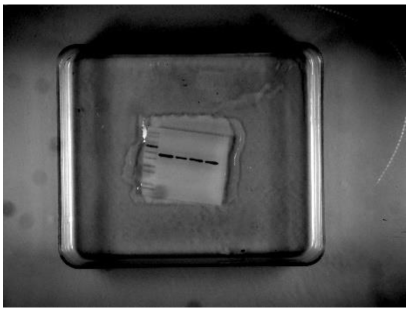

GAPDH

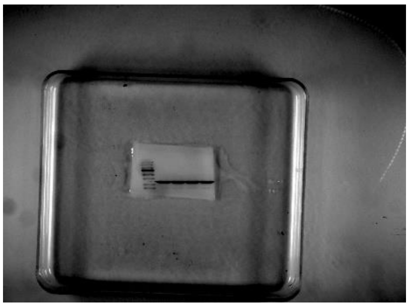

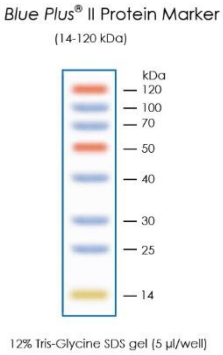

ACSL4

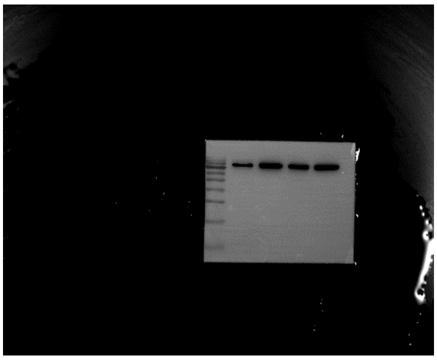

GAPDH

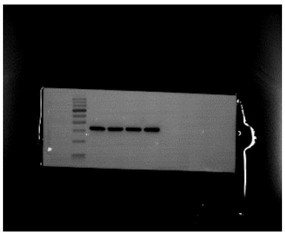

Supplement: Supplementary file 1 — Supplementary Figures. [file 41598_2024_70926_MOESM1_ESM.pdf]
